# Black Phosphorus Nanoparticles Promote Osteogenic Differentiation of EMSCs Through Upregulated TG2 Expression

**DOI:** 10.1186/s11671-021-03610-2

**Published:** 2021-10-12

**Authors:** Naiyan Lu, Xinhe Wang, Wentao Shi, Lu Bian, Xuan Zhang, Guofeng Yang, Xue Tang, Jun Wang, Yin Zou, Yuyan Weng

**Affiliations:** 1 State Key Laboratory of Food Science and Technology, Jiangnan University, Wuxi, 214122 Jiangsu Province People’s Republic of China; 2 The Affiliated Wuxi Children’s Hospital of Nanjing Medical University, Wuxi, 214023 Jiangsu Province People’s Republic of China; 3 School of Medicine, Jiangnan University, Wuxi, 214122 Jiangsu Province People’s Republic of China; 4 Center for Soft Condensed Matter Physics and Interdisciplinary Research, Soochow University, Suzhou, 215006 People’s Republic of China

**Keywords:** Black phosphorus nanoparticles, EMSCs, Osteogenic differentiation, Transglutaminase 2

## Abstract

At bio-safe concentrations, black phosphorus nanoparticles activated TG2, and promote the expression of ECM, which further promoted osteogenic differentiation of EMSCs. From these results, we can conclude that black phosphorus nanoparticles are suitable as biological factors in bone tissue engineering. Black phosphorus nanoparticles (BPs) present excellent biocompatibility and good biodegradability, which have been rigorously studied and proven. However, its utilization in bone tissue engineering fields is still in its infancy. Thus, the main purpose of the present study was to investigate the effects of BPs on osteogenic differentiation of ectodermal mesenchymal stem cell (EMSC) in vitro. Biocompatible BPs with high yield were prepared with a simple and efficient ultrasonication technique. EMSCs were isolated from adult rat nasal respiratory mucosa. Then, we treated EMSCs with BPs at different concentrations in vitro and examined the effect of BPs on osteogenic differentiation of EMSCs. In addition, inhibitor of transglutaminase 2 (TG2) and western blot were used to clarify the mechanism of the promoting effect of BPs on osteogenesis. Our results indicated that BPs could significantly enhance osteogenic differentiation of EMSCs in vitro. Nevertheless, BPs had no effect on EMSCs proliferation. Mechanistically, BPs promoted osteogenesis differentiation of EMSCs through upregulating TG2 expression. These results highlight the advantage of using chemical materials for novel engineering strategies of these highly promising small molecules for bone-tissue regeneration.

## Introduction

In clinical settings, lack of bone regeneration may result in a poor prognosis even in common bone fractures, and an urgent need for bone regenerative materials exists [1]. Subsequently, many therapeutic strategies, such as autografts, allografts, and artificial bone scaffolds have been used as regenerative materials. In recent years, biomaterials have successfully been demonstrated to contribute to bone regeneration, which shows the impressive progress in diversified biomaterial applications [2]. However, development of an artificial bone substitute with excellent osteo-conduction, osteo-induction, and osteo-integration is still urgently needed. Bioactive polymers [3–5] and ceramics [6] have been made into scaffolds for bone engineering, and scaffolds enhancing osteogenesis by releasing ions [7] are of particular concern. Notably, inorganic phosphates that specifically act on target cells or tissues can provide a promising path for studying mineralization-related biological processes and advancing bioinspired mineralization-guided medical engineering [8].

In 2014, Li and co-workers reported that black phosphorus (BP) nanosheets could be exfoliated from bulk BP and demonstrated the potential of BP nanosheets as a new two-dimensional (2D) material for applications in nano-electronic devices [9]. Due to BP’s superior properties, such as distinct pleated structures in layers, an adjustable direct band gap, high carrier mobility, and many interesting in-layer anisotropies, BP is currently under investigation for potential biomedical applications [10]. Owing to those advantages, BP nanomaterial-based scaffolds to stimulate bone regeneration have been extensively investigated in past 2 years. Previous studies have shown that phosphorus-based biomaterials could enhance mineralization and bone regeneration by increasing the local concentration of phosphate ions [11]. Although BP could be considered an ideal candidate to promote bone regeneration, it is commonly encapsulated into polymers or introduced onto a biomaterial scaffolds via immersion for application for a bone tissue engineer. Until now, the molecular mechanisms that modulate bone regeneration by BP remain largely unknown and thus hinders further development of BP-based therapies for bone repair in the clinic.

Mesenchymal stem cells (MSCs) are multipotent stromal cells with that have the capabilities of undergoing self-renewal and multi-lineage differentiation [12]. After bone fractures, MSCs take on a key role in the in vivo bone repair process [13–15]. The bone marrow stem cells (BMSCs) and their potential of osteogenic differentiation have been wildly studied over the years. However, the harvest process of BMSCs could be painful for the providers, with risks of infection. Originating from the cranial neural crest during embryonic development, ectodermal mesenchymal stem cells (EMSCs) can be easily harvested from the respiratory mucosa of the nasal cavity in adults. Besides, EMSCs are self-renewable with multidirectional differentiation potential, which have been thoroughly characterized in our previous studies [16, 17]. We have proved that EMSCs can differentiate into several cell lineages including adipocytes, neurocytes, chondrocytes and osteocytes [18, 19]. These special properties render EMSCs as a promising tool for exploring potential molecular mechanisms that modulate the osteogenic differentiation of EMSCs by chemical signals, including BPs. However, osteogenic differentiation is a complex process involving tight coordination of proliferation and differentiation of different cells, synthesis, and mineralization of the extracellular matrix (ECM) [20]. Previous studies have reported that transglutaminase 2 (TG2) is capable of stimulating osteoblast osteogenesis by influencing osteoblast proliferation, differentiation, extracellular matrix production, and mineralization [21–23].

The reports cited above indicate that BPs possess a tremendous potential for biomedical applications that could be a superior osteogenic differentiation inducer for EMSCs. Until now, research regarding the effects of BPs on the EMSC differentiation has not been reported. The main purpose of the present study was to investigate the effects of BPs on EMSC-associated in vitro osteogenic differentiation and proliferation. After that investigation, the potential molecular mechanism of BPs on EMSC proliferation and osteogenic differentiation was also carefully examined. Overall, our data show that BPs could be a potentially useful chemical agent for the tissue-engineered scaffolds.

## Materials and Methods

### Black Phosphorus Nanoparticles

Bulk BP was purchased from by Nanjing XFNANO Materials Tech Co., Ltd. Dimethyl sulfoxide (DMSO), sodium hydroxide (NaOH), and *N*-methyl-2pyrrolidone (NMP) were provided by Aladdin Industrial Co., Ltd. Phosphate-buffered saline (PBS) was obtained from Beijing Solarbio Science & Technology Co., Ltd. Dulbecco's modified Eagle's medium/nutrient mixture F12 (DMEM/F12; Hyclone; GE Healthcare Life Sciences), streptomycin, penicillin, fetal bovine serum (FBS), were obtained from BI Science and Technology Co., Ltd.

BP synthesis was similar to that in previous reports with minor modifications [24]. First, 20 mg bulk BP was immersed in saturated NaOH/NMP solution and refined with a grinding mechanical method. The mixture was then centrifuged for 10 min at 1500 rpm after which the precipitate was discarded. Afterward, the mixture was sonicated for 6 h in an ice/water bath, and the mixture was then filtered through a 100 μm BD Falcon filter (Becton Dickinson, Sunnyvale, CA). Finally, further centrifugation of suspension (10 min at 13,000 rpm, 4 °C) was performed, and the precipitate was collected.

### Immunofluorescence Staining of the EMSCs in the Nasal Mucosa

In order to show the EMSCs in vivo, the respiratory mucosa of the nasal septum was dissected and then fixed in 4% PFA overnight for immunofluorescence staining. The tissues were washed with PBS and then dehydrated with gradient sucrose solutions. The tissues were embedded in OCT (Sakura Finetek, Japan) for cryosection and were cut into coronal serial sections at a thickness of 25 mm using a Leica cryo-microtome. These sections were washed in PBS for 10 min and permeabilized with 1% bovine serum albumin and 0.1% Triton-X 100 in PBS. EMSCs in the nasal mucosa were immunofluorescence stained with the primary antibody of anti-nestin and the Cy3-conjμgated secondary antibody. The parallel negative controls were subjected to the same procedures without primary antibodies. The stained tissues were observed under an immunofluorescence microscope (AxioObserver, ZEISS, Germany).

### Cell Culture

All of the animal procedures were approved by the Jiangnan University Animal Care and Ethics Committee, and the International Guidelines for Animal Research were strictly followed in this study. Primary ecto-mesenchymal stem cells were isolated from rat respiratory mucosa according to our previous studies [17, 25]. Briefly, 100 g adult Sprague Dawley (SD) rats were anesthetized with intraperitoneal injections of pentobarbital sodium (0.05 g/kg). The middle third of the nasal septum was minced and then incubated in a 0.25% trypsin solution (Hyclone; GE Healthcare Life Sciences) at 37 °C for 25 min after which the nasal septum mucosa was carefully stripped. Finally, the nasal septum mucosal tissue was cut into pieces. The resulting suspension of the tissues was passed through a 100-um nylon mesh sieve, centrifuged at 1000 g for 5 min and then washed twice with phosphate-buffered saline (PBS). The tissue suspension was placed into a cell culture flask in growth medium (DMEM/12 contain 10% FBS, 100 U/ml penicillin, and 100 μg/ml streptomycin) and cultured at 37 °C in 5% CO_2_ and 95% air with saturated humidity. The medium was replaced every three days, and the cells were passaged every week. Cells in their third passage were used for all of the characterization studies. Immunofluorescence was carried out to characterize the cultured cells with antibodies against stem cell markers including vimentin and nestin [26].

### Identification of Multidirectional Differentiation Ability of EMSCs

The EMSCs were seeded in the 6-well plates and cultured with DEME/F12 medium for 24 h to 70% confluence. The medium was then changed with osteogenic differentiation medium (DMEM supplemented with 10% FBS, 0.1 mM dexamethasone, 10 mM β-glycerophosphate disodium, and 0.2 mM l-ascorbic acid) to induce osteogenesis. The medium was changed every seven days and Alizarin Red S staining was performed with 0.5% Alizarin Red S (Sigma-Aldrich) at day 28. EMSCs grown into 90% confluence were cultured in adipogenic differentiation medium to induce adipogenesis for 21 days. Oil red staining was conducted with Oil red staining kit (Solarbio) according to manufacturer’s instructions.

### Characterization of BPs

Scanning electron microscopy (SEM) was used to characterize BP morphology and size. Samples were prepared by placing a drop of the BP solution at a concentration of 1 mg/ml in deionized water onto a formvar-coated copper grid and then air drying. Samples were photographed by scanning electron microscopy (H-7500; Hitachi, Tokyo, Japan). The mean size of the BPs was analyzed using image Pro Plus software (Media Cybernetics Inc., MD, USA). The evaluation of zeta potential was confirmed by Malvern zeta sizer (ZEN3600). X-ray diffraction (XRD) was performed with a Bruker D8 Discover diffractometer in a Bragg–Brentano para-focused geometry and Cu Kα radiation. Diffraction patterns were collected between 10° and 80° of 2*θ* with a step of 0.01° 2*θ* and acquisition time of 0.2 s per step. The resulting data were evaluated using HighScore Plus 3.0e software. Raman spectrometer (LabRam HR800) with 514 nm laser excitation was used to measure the Raman spectra of sample. The surface composition of sample was measured through X-ray photoelectron spectroscopy ([XPS] Omicron NanoTechnology GmbH, Germany).

### Cell Counting Kit‑8 (CCK‑8) Assay

The effect of BPs on cell proliferation was evaluated using the cell counting kit (CCK-8) assay. Briefly, EMSCs (3000 cells/well) were plated in 96-well plates and treated with BPs (0, 1, 2, 4, 8, 16, 32, 64, 128, and 512 μg/ml) for 24 h at 37 °C. For CCK8 detection, 10 μl CCK8 reagent was added to the culture medium 4 h before analysis. Optical density (OD) at 450 nm was measured using a microplate reader (Thermo Fisher Scientific, Madrid, Spain) according to the manufacturer’s instructions. The concentration of BPs used for further investigation was selected based on the results of the CCK-8 assay. As for Ki-67 staining, EMSCs (1 × 10^4^) were cultured in 24-well plates and stained by immunofluorescence for Ki-67 (rabbit polyclonal; cat. no. ab15580; abcam; 1:300). DAPI was used to stain the nuclei. Images were captured by fluorescence microscopy (magnification, 200×; eclipse Ti; nikon corporation) and were analyzed with Image Pro Plus software.

### Osteogenic Differentiation

For osteogenic differentiation, EMSCs were seeded at a density of 3,000 cells/cm^2^ and cultured in growth medium to 70% confluence. Cells were then induced with osteogenesis differentiation medium for 2 weeks. Notably, in order to avoid the influence of β-Sodium glycerophosphate, the osteogenesis differentiation medium was supplemented with 10% FBS, 0.1 mM dexamethasone, and 0.2 mM l-ascorbic acid (Sigma-Aldrich (St. Louis, MO, USA) but not with β-Sodium glycerophosphate. After inducing differentiation, alkaline phosphatase (ALP) and Alizarin red staining and RT-qPCR were used to assess for any effects of BPs on osteogenic differentiation of EMSCs. EMSCs were seeded at a density of 2 × 10^5^ cells/well in 6-well plates and incubated with osteogenic medium. According to the manufacturer's instructions, ALP staining was assessed using an ALP staining kit (Nanjing Jiancheng Bioengineering Institute) on day 14. Alizarin red S staining was applied to visualize the deposition of calcium phosphate. Briefly, the fixed cells were washed again with deionized water and incubated with 1 ml/well of Alizarin red staining solution (0.5% (w/v) Alizarin Red S (Sigma-Aldrich) in PBS for 10 min at 37 °C. After the solution was removed, the sample was washed again with deionized water. Positive staining area indicating the calcified nodules per field was counted with Image-Pro plus software and normalized to respective control.

### RT-qPCR

RT-qPCR was performed as previously described. Briefly, total RNA was isolated from mono-cultured or sorted using the RNA purification kit (TaKaRa, Japan), according to the manufacturer’s instructions. Two micrograms of mRNA were reverse-transcribed into cDNA using a cDNA synthesis kit (Fermentas). Primers used for RT-PCR were designed and constructed by Nanjing GenScript Bioengineering Technology and Services Co., Ltd (Nanjing, China), shown in Table 1. RT-PCR was manipulated using a SYBR Green/Fluorescein qPCR Master Mix kit (Fermentas) with the ABI Prism 7500 system. The data were normalized to glyceraldehyde 3-phosphate dehydrogenase (GAPDH) to indicate relative expression levels. All measurements were performed in triplicate.

**Table 1:** Primers used for quantitative reverse transcription polymerase chain reaction

Gene	Forward primer sequence (5–3)	Reverse primer sequence (5–3)
RUNX2	TCATGGCCGGGAATGATGAG	CGCTCCGGCCTACAAATCTC
TG2	CTCAATTGGCATCAAAAAGGGC	AGCCTCCTTCCTTTGGTAGC
OPN	TGTGAAACTCGTGGCTCTGA	GAACCAAGCGTGGAAACACA
OCN	ACAGTGACCTGAGTGAGGGT	GGGACTCCTGGCTGTTCATC
BMP-2	CGTTGATCAAATCAGAAGCCAT	AGCTCCGCAGATGTGAGAAA
COL-1	CCTGGTGCTGATGGACAACC	TTTTAGCCCAAGGGTGAAGGG
GAPDH	ACATTGGGGGTAGGAACACG	GCCATCACTGCCACTCAGAA

### Western Blot

The cultured cells were washed twice with PBS and then lysed on ice for 30 min with RIPA lysis buffer containing a phosphatase and protease inhibitor cocktail. To collect the total TG2, the cell lysates and ECM in the 6-well plates were collected by a cell scraper. The plates were washed with PBS, and the cell-wash fluids were also collected. The lysates were harvested from the supernatant by centrifugation at 12,000 × *g* for 5 min, mixed with an equal volume of SDS loading buffer, and then boiled for 5 min. Protein concentrations were determined using a BCA Protein Assay kit (Beyotime, Shanghai, China, P0012). Proteins were extracted in RIPA lysis buffer, separated on sodium dodecyl sulfate (SDS) polyacrylamide gels, and electrophoretically transferred onto nitrocellulose membranes (Millipore, LA, USA). Membranes were blocked with 1% bovine serum albumin (BSA) in 0.01 M Tris-buffered saline (TBS) containing 0.5% Tween 20 (Suolaibao, Beijing, China) and blotted with the indicated antibodies, including anti-TG2 (1:200; sc-48387, Santa Cruz Biotechnology, Inc.), anti-FN (1:500; cat. no. BA1772; Wuhan Boster Biological Technology, Ltd.) and anti-LN (1:500; cat. no. BM4921; Wuhan Boster Biological Technology, Ltd.), anti-OCN(1:200; sc-390877, Santa Cruz Biotechnology, Inc.), anti-OPN (1:200; sc-73631, Santa Cruz Biotechnology, Inc.), anti-COL I (1:500; cat. no. BA0325; Wuhan Boster Biological Technology, Ltd.), at 4 °C for 12 h. The membranes were then reacted with horseradish peroxidase-conjugated goat anti-rabbit-IgG (1:5000; Boster, Wuhan, China) for 2 h at room temperature. The protein bands were visualized using a Pierce ECL Plus substrate (Thermo Fisher Scientific) and then scanned with a Chemiluminescence Imaging System (Clinx Science Instruments, Shanghai, China).

### Inhibition Experiments

To confirm the involvement of TG2, TG2 inhibition experiments were carried out on EMSCs. Neutralization experiments were performed with an anti-TG2 neutralizing antibody (from BD Biosciences, San Diego, CA). Cells were seeded in 6-well plates with normal culture medium for 24 h to adhere, and were treated with anti-TG2 antibody (10 μg/ml). At the same time, the control group was set. After being seeded into 6-well plates for 24 h, EMSC osteogenesis was induced for 14 days using osteogenic medium with BPs (2 μg/ml), and the culture medium containing inhibitors was changed by fresh osteogenic medium every 7 days. Alizarin red S staining was applied to visualize the deposition of calcium phosphate. Western blotting was used to assess for levels of osteocalcin, osteopontin, and collagen type 1 (OCN, OPN, and COL I expression, respectively).

### Immunofluorescence Staining

The cultured cells were fixed with 4% paraformaldehyde for 8 h at 4 °C. The fixed cells were washed three times with PBS and permeabilized for 30 min in PBS containing 0.2% Triton X-100 and 1% BSA (Suolaibao, Beijing, China). After permeabilization, these fixed cells were washed with PBS and then incubated with primary antibodies at 4 °C for 8 h followed by removal all of the primary antibodies. Cells were then washed three times with PBS and incubated for 2 h at room temperature with secondary antibodies Alexa Fluor-594 (1:800, Life Technologies Molecular Probes, Carlsbad, CA, USA) at 37 °C for 2 h. The nuclei were counterstained with 4′6-diamidino-2-phenylindole ([DAPI]; Sigma-Aldrich). All of the tested groups were observed under an immunofluorescence microscope.

### Statistical Analysis

Data were obtained from the three separate experiments described above and presented as mean ± standard deviation (SD). Analysis of the data distribution was performed using the student’s *t*-test to analyze the significance of differences between the treated and control groups. One way analysis of variance (ANOVA) followed by the Tukey post-hoc test was performed to assess the significant differences between groups. *p* < 0.05 was considered statistically significant.

## Results

### Characterization of BPs

**Fig. 1:**
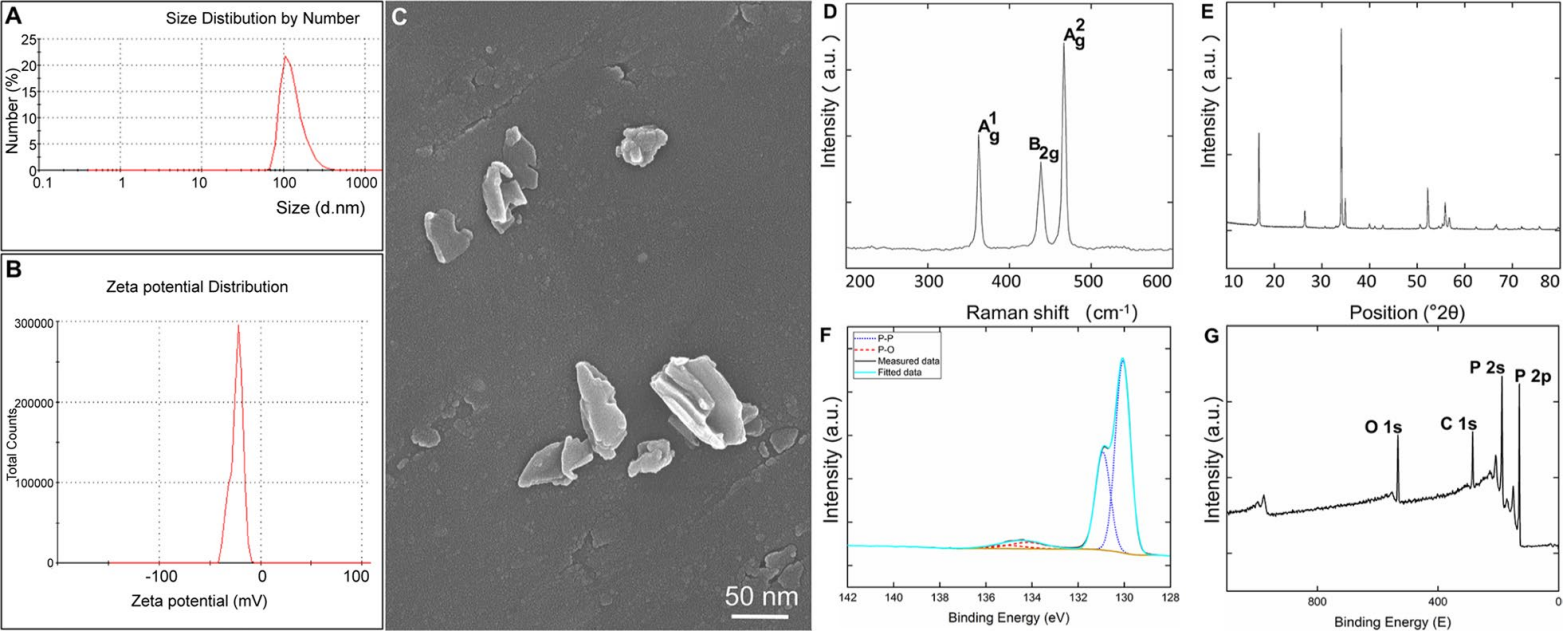
Characterization of BPs. **A** Size distribution of black phosphorus nanoparticles (BPs); **B** zeta potential report of BPs with a zeta potential of − 23.7 ± 0.65 mV; **C** scanning electron microscopy (SEM) images of the BP nanosheets (BPNs). **D** Raman spectra of BPs; **E** X-ray diffractogram and **F** P 2p high-resolution X-ray photoelectron spectroscopy (XPS) spectra of BPs; **G** XPS spectra of BPs

The BP size distribution measured by dynamic light scattering (DLS) was relatively narrow in the range of 100 to 200 nm with a peak value at 132 nm. The result is shown on Fig. 1A. The zeta potential of BPs was determined as − 23.7 ± 0.65 mV (Fig. 1B), confirming the stability of the BPs. The size of particles was further investigated by scanning electron microscopy (SEM) (Fig. 1C). The results correspond well with the measurement of particle size distribution by DLS.

Raman scattering (Fig. 1D) revealed three prominent peaks at 362.3, 438.5, and 466.9 cm^−1^ associated with the out-of-plane phonon mode (*A*^1^_g_) and two in-plane modes (*B*^2^_g_ and *A*^2^_g_) of BP, [17, 18], respectively. XRD (Fig. 1E) shows the phase purity of the prepared material, with an average crystalline size of 102.7 nm. Broadening of the diffraction pattern indicates the nanometer size of individual crystallites. High-resolution XPS spectra of P 2 p (Fig. 1F) show the main peak at 130 eV corresponding to the P–P bond of black phosphorus in addition to an additional peak at 135 eV originating from P–O bonds caused by surface oxidation of the BPs. BP surfaces were examined by wide-scan X-ray (Fig. 1G).

### Characterization of EMSCs

**Fig. 2:**
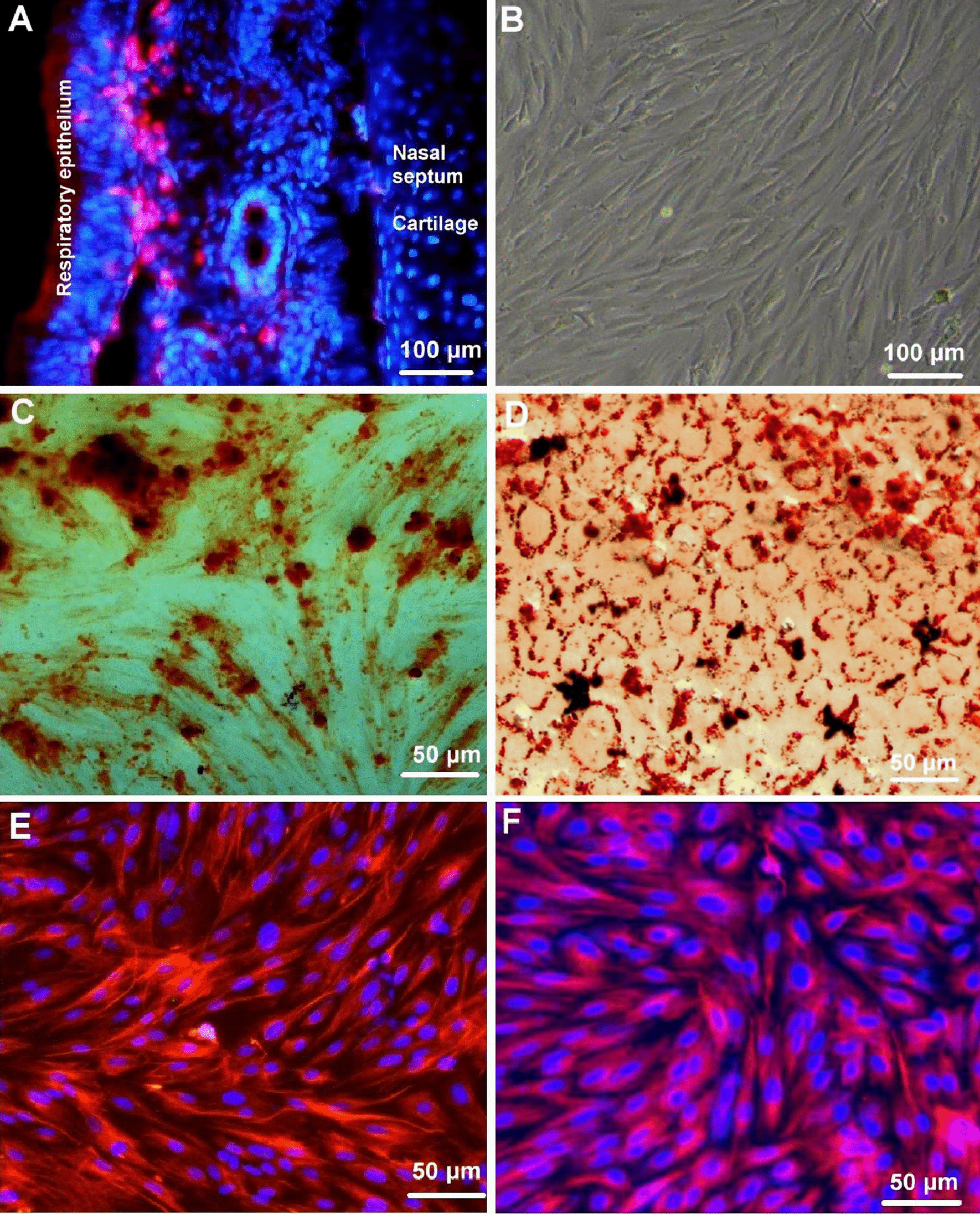
Identification of the ectodermal mesenchymal stem cells (EMSCs). **A** The nestin positive EMSCs (cy3, red) were located in the lamina propria under the respiratory epithelium of the nasal septum; **B** A phase-contrast image showed that the cultured EMSCs at the third passage appeared as fibroblastic-like cells and proliferated rapidly on plastic plates; **C** The osteogenic differentiated EMSCs in osteogenic medium were stained by alizarin red; **D** The adipogenic differentiated EMSCs in adipogenic-induction medium were stained by with oil red-O; **E**, **F** Immunofluorescence staining of the neural crest cell markers showed that almost all of the EMSCs expressed nestin and vimentin. The IgG-Cy3 (red) was used as the second antibody for the immunofluorescence staining and the nuclei were counter-stained with Hoechst 33342 (blue)

The immunofluorescence staining of the nasal mucosa showed that the nestin-positive EMSCs were located in the lamina propria under the respiratory epithelium of the nasal septum (Fig. 2A). The cultured EMSCs at the third passage appeared as fibroblastic-like cells and proliferated rapidly on plastic plates (Fig. 2B). We evaluated osteogenic differentiation in osteogenic medium by alizarin red staining on day 28 (Fig. 2C). The adipogenic differentiation in adipogenic induction medium was assessed at day 21 by staining with oil red-O solution (Fig. 2D). Immunofluorescence staining showed almost all of the EMSCs expressed neural crest cell markers, such as nestin (> 95%) as shown in Fig. 2E and vimentin (> 95%) as shown in Fig. 2F. The results for stem cell markers’ co-expression indicate that these EMSCs are a type of mesenchymal stem cell.

### Cytotoxicity

**Fig. 3:**
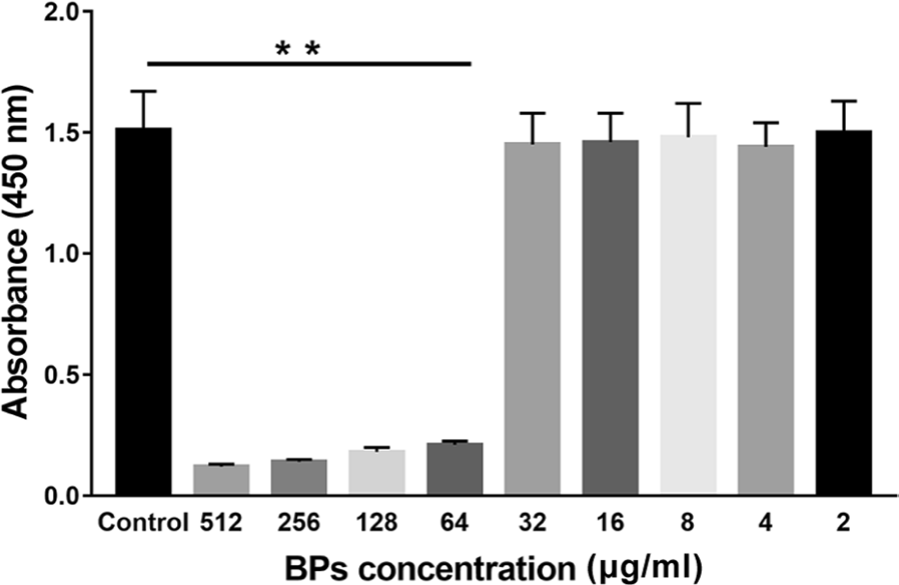
The cytotoxicity of BPs. Cells were treated with BPs (0, 2, 4, 8, 16, 32, 64,128, 256, and 512 µg/ml) for 24 h, and EMSC viability was tested by cell counting kit (CCK-8) proliferation assay (*n* = 9). Data were expressed as mean ± standard deviation (SD). **p < 0.01.

**Fig. 4:**
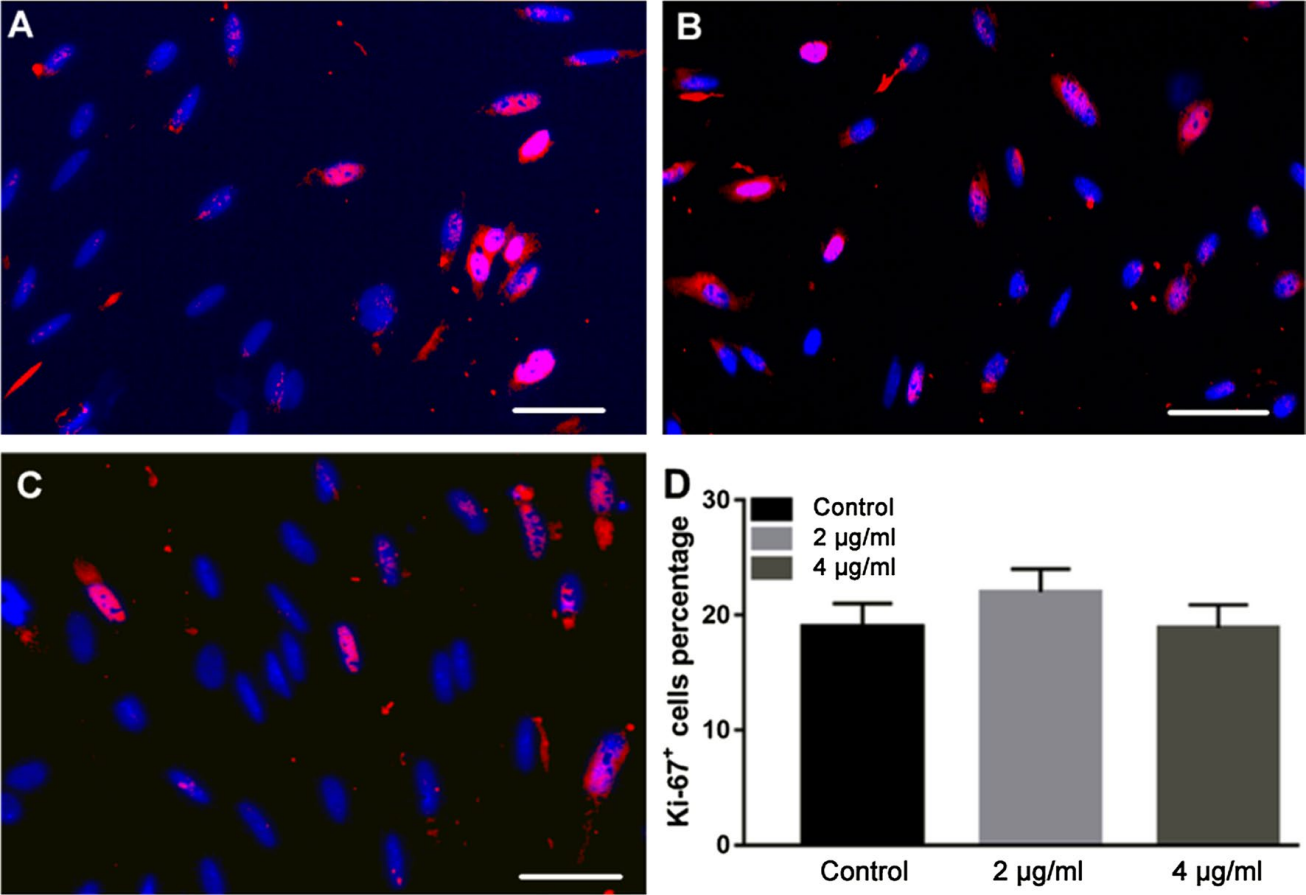
The evaluation of cell proliferation of the BPs-treated group. EMSCs were treated with BPs (0, 2, and 4 μg/ml) for 24 h. Cell proliferation was measured by immunofluorescent staining with anti-Ki67 antibody (red) and DAPI (blue), and merged images were shown (**A**–**C**). Ki67-positive cells among 4′,6-diamidino-2-phenylindole (DAPI)-positive cells were counted in two high-power fields in each of three plates. Data are expressed as the mean ± SD (**D**).

As a preliminary experiment, CCK-8 assay was performed to assess the cytotoxicity of BPs. The viability of EMSCs after 24 h of exposure to the BPs is shown in Fig. 3. No significant cytotoxicity after exposure to up to 32 μg/ml of BPs was noted. However, exposure to BPs causes significant cytotoxicity at higher doses (> 32 μg/ml). Ki-67 expression in the nucleolus and chromosomes was observed in the low doses group, (Fig. 4A–C). However, the ratio between the Ki-67 positive nuclei and the total population of nuclei showed no significant difference in low dose BP-treated EMSCs compared to untreated controls (Fig. 4D). Therefore, in the present study, we choose concentrations of 2 and 4 μg/ml of BPs in the following experiments in which significant cytotoxicity was not observed.

### Alizarin Red S Staining

**Fig. 5:**
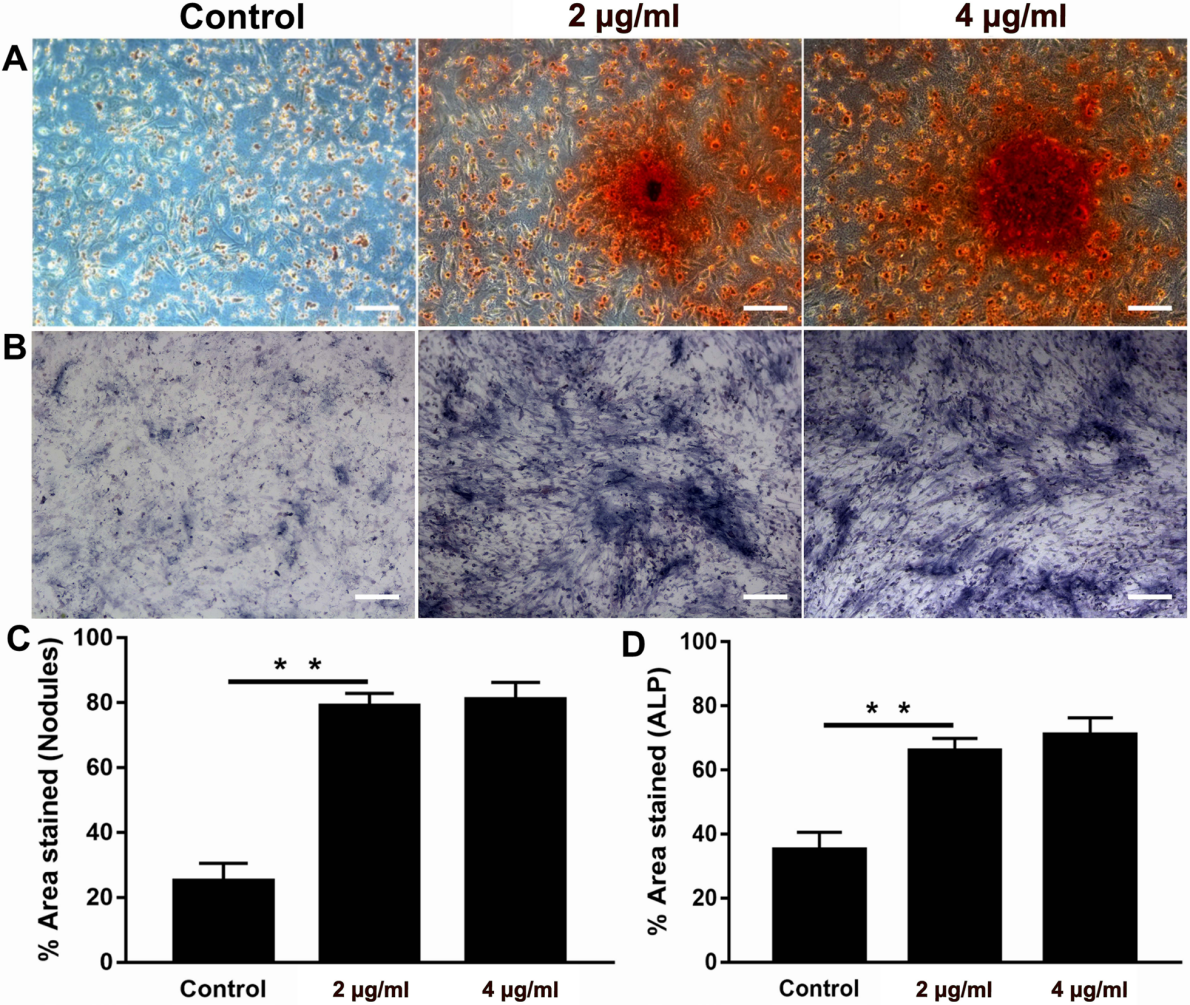
Alkaline phosphatase (ALP) and Alizarin Red staining assays. **A** Osteogenic-differentiated EMSCs stained with Alizarin Red solution at day 14; **B** ALP staining was visualized under microscope; **C** the chart shows the quantification of Alizarin red deposition areas; **D** significantly higher ALP activity was shown in the BP-treated EMSCs group than that of the control group. Data were expressed as the mean ± SD. **p < 0.01

As shown in Fig. 5A, mineralization in the cells was evaluated by Alizarin red S staining after being treated with the conditioned medium for 14 days. Calcium nodules were all observed in three groups, while unshaped calcium nodules were present in the control group. Compared with the control group, the calcium nodules deposited in the 2 and 4 μg/ml groups were larger (*p* < 0.05), but no obvious differences were observed between the treatment groups (*p* > 0.05) as shown in Fig. 5C.

### ALP Tests

ALP staining was conducted to study the osteogenic differentiation of EMSCs. Compared with those in the control group, the EMSCs in the 2 and 4 μg/ml BP groups were darker in color (Fig. 5B), suggesting that the addition of BPs caused enhancement of ALP expression in EMSCs. The cells treated with BPs had higher ALP activity than the control group on day 7 (*p* > 0.05), but no significant difference between 2 and 4 μg/ml was observed (*p* > 0.05) as shown in Fig. 5D.

### RT-qPCR

**Fig. 6:**
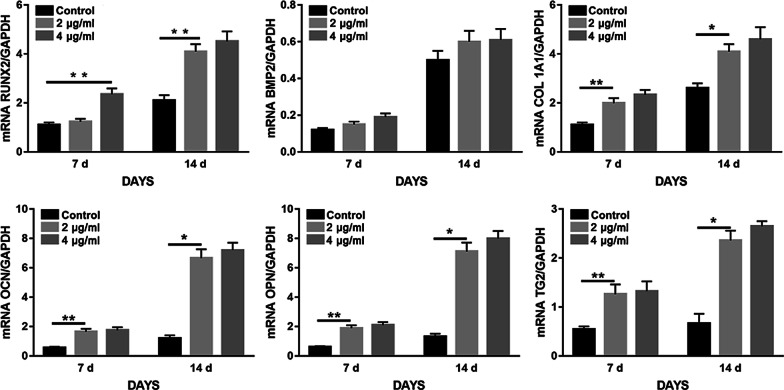
BPs potentiates osteogenesis of EMSCs. EMSCs were grown in osteogenic medium with 0 μg/ml, 2 μg/ml, 4 μg/ml BPs 14 days. Expression of genes involved in osteogenesis of EMSCs was quantified by quantitative reverse transcriptase polymerase chain reaction (RT-qPCR). **p < 0.01, *p < 0.05.

The main differentiation markers, including runt-related transcription factor 2 (RUNX 2), ALP, COL 1, OCN, OPN, and bone morphogenic protein 2 (BMP-2), were analyzed on day 14 as shown in Fig. 6. Expression of all of these gene markers was found in the three groups of cells on day 7. No obvious differences in gene expression between 2 and 4 μg/ml groups were found. However, compared with control cells, expression of the above-described genes in BP-treated cells was significantly increased.

### BPs Enhances Osteogenesis of EMSCs by Up-Regulating TG2 Expression

**Fig. 7:**
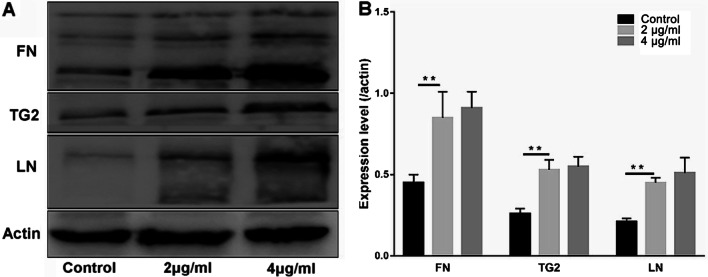
Transglutaminase 2 (TG2) was involved in the osteogenic differentiation of EMSCs. EMSCs were incubated with BPs (2 and 4 μg/ml) for 14 days, and fibronectin (FN), LN, and TG2 levels were assessed by immunoblotting. Data were expressed as mean ± SD. **p < 0.01; *p < 0.05.

**Fig. 8:**
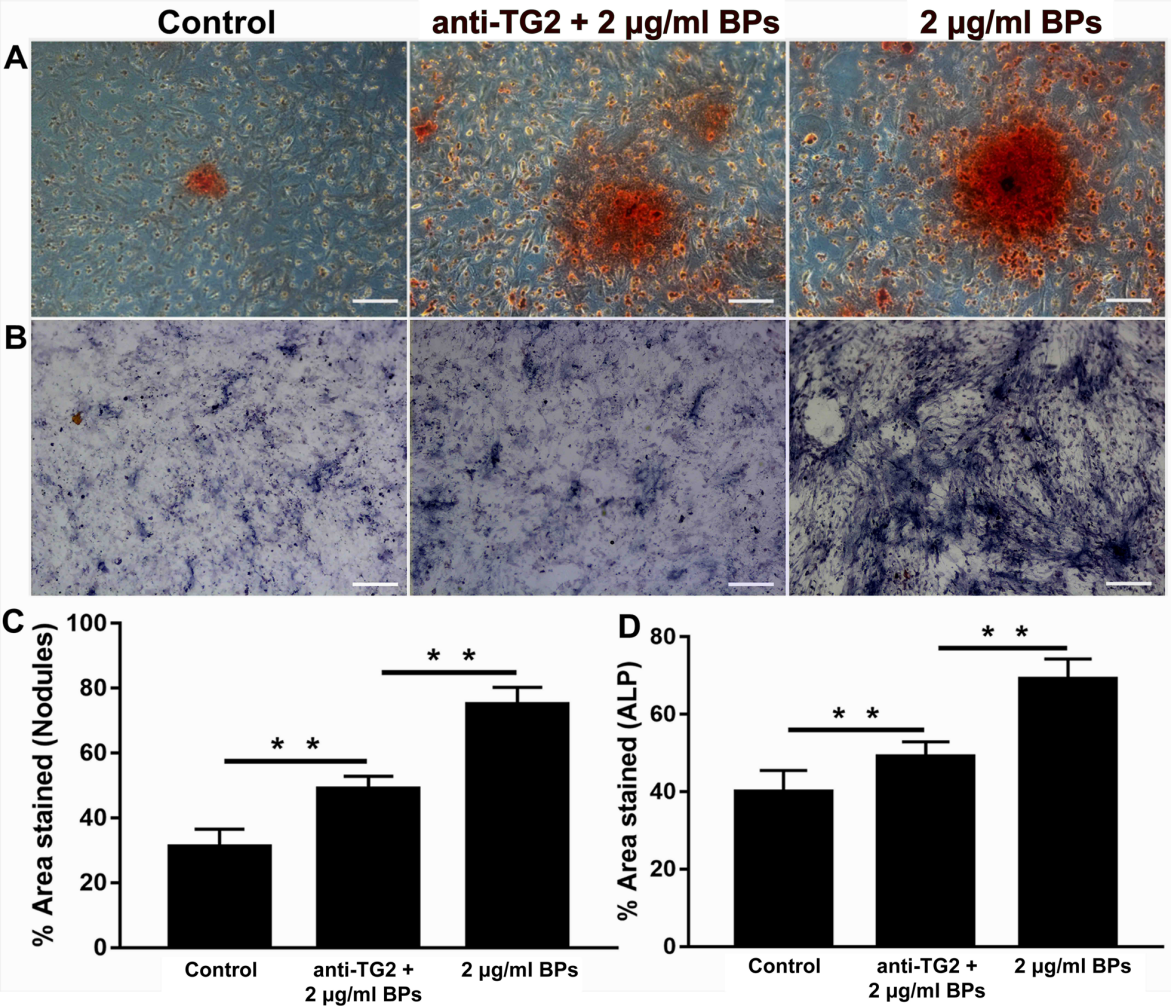
Osteogenic differentiation of EMSCs with TG2 neutralizing antibody. **A** ALP staining. **B** ARS staining performed to examine extracellular mineralization. **C** Quantitative ALP analysis. **D** Quantitative Alizarin red staining analysis. **p < 0.01

**Fig. 9:**
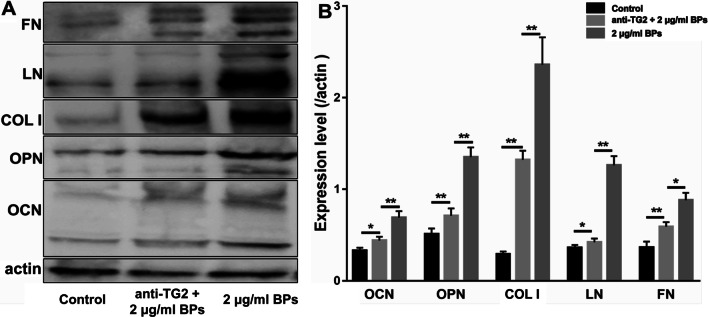
Anti-TG2 inhibited BP-induced EMSC osteogenic differentiation. **A** Representative western blots of osteoponin, osteocalcin, and collagen 1 (OPN, OCN, LN, FN, and COL I, respectively) in differentiated EMSCs following treatment with 2 μg/ml BPs and 2 μg/ml anti-TG2 or 2 μg/ml BPs alone. **B** Quantification of OPN, OCN, and COL I protein expression. Data were expressed as mean ± SD. **p < 0.01; *p < 0.05

Since a significant impact of TG2 on integrin-mediated differentiation and ECM deposition was demonstrated for a wide range of cells, we investigated whether BPs would promote osteogenic differentiation of EMSCs via upregulation of TG2 expression. As shown in Fig. 7, we detected obvious upregulation of the intracellular and extracellular TG2 in the BP-treated groups. Laminin (LN) and Fibronectin (FN) in the 2 and 4 μg/ml BP-treated cells were of higher levels than those in the control group (Fig. 7A). EMSCs exposed to BPs showed an approximately twofold increase in TG2 levels compared with the control group, but no significant difference was observed between the BP-treated groups (Fig. 7B). As a macro-molecular protein, the anti-TG2 antibody failed to cross the cell membranes. Thus, the extracellular TG2 was blocked by the antibody and failed to crosslink various growth factors and ECM proteins. The Alizarin Red S (Fig. 8A, C) results showed that anti-TG2 markedly suppressed the BP-mediated increase in calcium and ECM deposition. Meanwhile, as shown in Fig. 8D, anti-TG2 significantly inhibited BP-treated cells’ increase in ALP activity. Moreover, anti-TG2 effectively abolished the effects of BPs on the bone matrix proteins expression, including OCN, OPN, and COL I (Fig. 9).

## Discussion

To the best of our knowledge, modification of phosphorus was first exhaustively studied as early as 1914; unfortunately, these studies did not receive much attention for an entire century [27]. Phosphorus is one of the essential elements making up about 1% of the total body weight as a bone component in the human body [28], while most of the other materials cannot warrant such a natural biocompatibility. In 2014, BP was introduced as a new member of the 2D layered materials.

In this study, the BP nanoparticles were prepared on a large scale from bulk BP crystals by using an improved mechanical grinding and continuous ultrasound method. Compared with other methods, this ultrasound and mechanical grinding synthesis is facile and efficient and enables production of BPs on a large scale. For characterization of BPs, SEM was carried out to examine the morphology of BPs. SEM images illustrated that BPs were successfully synthesized, and the typical sizes of BPs are about 100 to 150 nm. The size of particles was further investigated using DLS in the relatively narrow range around 133 nm. The result of SEM corresponded well with the measurement of particle size measurements based on DLS. Interestingly, previous studies demonstrated that BP with larger lateral size has higher cytotoxicity than small BP, while ultra-small BPs were even considered nontoxic up to the high concentration of 1000 μg/ml [29, 30]. Thus, for biomedical applications, the BP nanomaterials still need further study. In addition, Raman scattering revealed the presence of three prominent peaks at 361.5, 437.1, and 465.2 cm^−1^ that were associated with the out-of-plane phonon mode (*A*^1^_g_) and two in-plane modes (*B*^2^_g_ and *A*^2^_g_) of BPs. These results were consistent with previous reports [31] in which showed that the BPs had been prepared successfully from bulk BP. XRD shows the phase purity of the prepared BPs. Broadening of the diffraction pattern indicates the nanometer size of individual crystallites.

To evaluate the stability and degradation rate of BPs, Wang and co-workers performed an experiment in which normalized absorption spectra of the BP nanosheets were dispersed in water and exposed to air. After 6 days, they found that the absorbance of the BP nanosheets at 450 nm decreased by 43% compared to the initial value, indicating that BP is easily degraded in the physiological environment [32]. Also, it is well-known that BP is sensitive to water and oxygen, but this shortcoming is considered a merit for biomedical applications. Because of these special characteristics compared with other biomaterials, BPs could potentially avoid material accumulation in human body and then reduce cytotoxicity caused by such material noumenon.

Since MSCs play key roles in bone formation, there is no doubt that transplanting MSCs in animal models of bone defects enhance bone regeneration and promotes functional recovery via BMSC acquisition [33]. Unfortunately, BMSC acquisition procedures are painful for the donor and frequently cause surgical site infection, and the number of harvested BMSCs is low [34]. Previous studies from our laboratory and other reports have shown that EMSCs could be isolated from several adult tissues, such as dental pulp and the nasal mucosa, without causing invasive injury. Moreover, EMSCs were easily induced into osteoblasts, rendering those cells as promising seed cells for bone tissue engineering. Therefore, EMSCs were used in this study. We attempted to obtain the maximum safety BP concentration for EMSCs. As shown in the results, we achieved the maximum safe concentration of BPs (less than 64 μg/ml). The concentrations of 2 and 4 μg/ml of BPs were chosen for further study to avoid any possible potential toxic. The Ki-67 assay was also used to quantify and evaluate EMSC proliferation in these samples after treating these cells with BPs (2 and 4 μg/ml) for 24 h. We did not observe any statistically significant suppression of EMSCs growth in the case of BPs. Meanwhile, our results indicate that during treatment with safe concentrations of BPs, promotion of EMSCs proliferation also occurred.

Some reports have shown that the concurrent binding of BP and calcium ions may benefit osteogenic differentiation, thereby leading to enhanced bone regeneration [35, 36]. To investigate these effects, in vitro EMSCs exposed to BPs were used in osteogenic differentiation experiments. Interestingly, the results of the ALP test and Alizarin Red S staining show that exposure to BPs could promote rather than compromise osteogenetic differentiation of EMSCs compared to the control group. Similar findings have been reported in which phosphorus-rich materials can stimulate mineralization and bone regeneration. Co-expression of osteogenesis-related genes, including ALP, OPN, OCN, COL1, and RUNX2, in differentiated EMSCs was detected at days 7 and 14 by RT-qPCR. Indeed, the expression levels of these osteogenic genes significantly increased in differentiated EMSCs treated with BPs, also providing confirmation of the osteogenic potential of the BPs. Similar results were reported in which BP could induce both the proliferation and osteogenic differentiation of human pre-osteoblast cells. Together these results indicate that the BPs were able to induce osteogenic differentiation of EMSCs.

It can be asked, “How do BPs promote osteogenetic differentiation of EMSCs?” It is well accepted that phosphorus can capture Ca^2+^ in vivo to form calcium phosphate deposits that accelerate bone regeneration, while BP can be the resource of phosphorus ions [36]. Tong et al. believes that BP generate mild heat (40–42 °C), which causes up-regulation of heat shock protein (HSPs) expression and stimulates bone regeneration [37, 38]. Moreover, in this study, the upregulated activation of TG2 levels was also observed in BPs treatment groups. To the best of our knowledge, TG2 has important enzymatic and non-enzymatic functions at these locations in which it crosslinks various ECM proteins and modulates the interactions of cells with the ECM and soluble growth factors by non-covalent interactions with and regulation of integrins [39–41]. Obviously, the BPs can react strongly with oxygen and water and finally degrade to non-toxic phosphate in aqueous solutions, which is a crucial component of ATP. Nakano Y et al. reported that ATP can act as a significant phosphate (Pi) source for mineralization in MC3T3-E1 osteoblast cultures, indicating that ATP-hydrolyzing enzymes could induce mineral deposition [42]. They also found that TG2 could not only act as a phosphatase but could be involved in ATP hydrolysis in the osteoblast cultures thus further contributing to the elevation in Pi levels required for mineral deposition, which may be beneficial to EMSC energy metabolism. This process may be part of the contribution that BPs makes toward enhancement of EMSC osteogenic differentiation. On the other hand, thanks to TG2 bio-functions, a wide variety of ECM adhesion proteins, including LN, COL I, and FN, could maintain a stable state. Indeed, in the present study, we observed that ECM (FN, COL I, and LN) were significant higher in BP-treated EMSC groups. BPs provide a favorable ECM microenvironment for promoting greater osteogenic EMSC differentiation and proliferation.

Until now, no secretory signal sequences and hydrophobic or transmembrane domains have been clearly identified in TG2 because the protein is not localized in the endoplasmic reticulum (ER)/Golgi compartments [39, 43], and only few studies reported about these factors that control TG2’s secretion. Therefore, it remains unclear as to the exact mechanism of BP regulation of expression patterns of TG2 in the progress of EMSC osteogenic differentiation.

## Conclusion

In the present study, BPs were successfully fabricated using mechanical grinding and continuous ultrasound method. At bio-safe concentrations, BPs activated TG2, and promoted the expression of ECM, which further promoted osteogenic differentiation of EMSCs. From these results, we can conclude that BPs would be suitable for incorporation into tissue-engineered scaffolds that utilize EMSCs to repair bone defects. Although our research highlights the great potential of BPs in nano-biomedicine, large-scale preclinical and clinical studies concerning its safety are needed before any clinical applications are established.

## Data Availability

The datasets generated during and/or analyzed during the study are available from the corresponding author on reasonable request.

## Abbreviations

BPs: Black phosphorus nanoparticles
EMSCs: Ectodermal mesenchymal stem cells
TG2: Transglutaminase 2
2D: Two-dimensional
ECM: Extracellular matrix
DMSO: Dimethyl sulfoxide
NMP: *N*-Methyl-2pyrrolidone
PBS: Phosphate-buffered saline
DMEM/F12: Dulbecco's modified Eagle's medium/nutrient mixture F12
FBS: Fetal bovine serum
SD: Sprague Dawley
SEM: Scanning electron microscopy
XPS: X-ray photoelectron spectroscopy
CCK-8: Cell counting kit
OD: Optical density
RT-qPCR: Reverse transcriptase polymerase chain reaction
GAPDH: Glyceraldehyde 3-phosphate dehydrogenase
BSA: Bovine serum albumin
TBS: Tris-buffered saline
DAPI: 4′6-Diamidino-2-phenylindole
DLS: Dynamic light scattering
